# Antibacterial and antibiotic-resistance modifying activity of the extracts and compounds from *Nauclea pobeguinii* against Gram-negative multi-drug resistant phenotypes

**DOI:** 10.1186/s12906-016-1173-2

**Published:** 2016-07-07

**Authors:** Jackson A. Seukep, Louis P. Sandjo, Bonaventure T. Ngadjui, Victor Kuete

**Affiliations:** Department of Biochemistry, Faculty of Science, University of Dschang, Dschang, Cameroon; Department of Pharmaceutical Sciences, CCS, Federal University of Santa Catarina, Florianópolis, 88040-900 SC Brazil; Department of Organic Chemistry, Faculty of Science, University of Yaoundé 1, Yaoundé, Cameroon

**Keywords:** Antibacterial, Gram-negative bacteria, *Nauclea pobeguinii*, Efflux pumps, Multidrug resistant, Resveratrol

## Abstract

**Background:**

Multi-drug resistance of Gram-negative bacteria constitutes a major obstacle in the antibacterial fight worldwide. The discovery of new and effective antimicrobials and/or resistance modulators is necessary to combat the spread of resistance or to reverse the multi-drug resistance. In this study, we investigated the antibacterial and antibiotic-resistance modifying activities against 29 Gram-negative bacteria including multi-drug resistant (MDR) phenotypes of the methanol extracts from *Nauclea pobeguiinii* leaves (NPL), *Nauclea pobeguiinii* bark (NPB) and six compounds from the bark extract, identified as 3-acetoxy-11-oxo-urs-12-ene (**1**), *p*-coumaric acid (**2**), citric acid trimethyl ester (**3**), resveratrol (**4**), resveratrol *β-*_D_*-*glucopyranoside (**5**) and strictosamide (**6**).

**Methods:**

The broth microdilution method was used to determine the minimal inhibitory concentrations (MIC) and minimal bactericidal concentrations (MBC) of crude extracts and compounds as well as the antibiotic-resistance modifying effects of MPB and **4**.

**Results:**

MIC determinations indicate values ranging from 32-1024 μg/mL for NPB and NPL on 89.7 % and 69.0 % of the tested bacterial strains respectively. MIC values below 100 μg/mL were obtained with NPB against *Escherichia coli* ATCC10536, AG100 and *Enterobacter aerogenes* CM64 strains. The lowest MIC value for crude extracts of 32 μg/mL was obtained with NPB against *E. coli* ATCC10536. Compound **4** was active all tested bacteria, whilst **1**, **3** and **6** displayed weak and selective inhibitory effects. The corresponding MIC value (16 μg/mL) was obtained with **4** against *Klebsiella pneumoniae* KP55 strain. Synergistic effects of the combination of NPB with chloramphenicol (CHL), kanamycin (KAN) as well as that of compound **4** with streptomycin (STR) and ciprofloxacin (CIP) were observed.

**Conclusion:**

The present study provides information on the possible use of *Nauclea pobeguinii* and compound **4** in the control of Gram-negative bacterial infections including MDR phenotypes. It also indicates that NPB and **4** can be used as naturally occurring antibiotic-resistance modulators to tackle MDR bacteria.

**Electronic supplementary material:**

The online version of this article (doi:10.1186/s12906-016-1173-2) contains supplementary material, which is available to authorized users.

## Background

Bacterial multi-drug resistance (MDR) constitutes a major impediment to antibiotherapy worldwide. Over-expression of tripartite efflux pumps of resistance–nodulation–cell division (RND) family such as AcrAB-TolC in enterobacteria or MexAB-OprM in *Pseudomonas aeruginosa* have been reported as one of the major mechanism of MDR in Gram-negative bacteria [1, 2]. High rates of resistance of Gram-negative bacteria to commonly used antibiotics has been previously reported in Cameroon [3]. Medically important enterobacteria over-expressing efflux pumps include various species such as *Escherichia coli, Klebsiella pneumoniae, Enterobacter aerogenes, Enterobacter cloacae, Providencia stuartii, Salmonella typhi* [4, 5]. The scarcity of the development of new antibiotics propels development of alternative medicine including phytotherapy. In fact, medicinal plants represent a good source of antimicrobials, in regards to the diversity of their secondary metabolites [6, 7]. African flora is very rich and has shown a good potential to fight various human ailments [8]. Therefore, exploring African flora for antibacterial drug discovery appears as an attractive strategy. In the past, several medicinal plants of the continent showed good antibacterial activities against MDR Gram-negative (MDRGN) bacterial species. Some of the most prominent plants include *Dichrostachys glomerata, Beilschmiedia cinnamomea, Combretum molle* [9, 10], *Piper nigrum* and *Telfairia occidentalis* [11], *Beilschmedia acuta* [12] and *Dorstenia psilurus* [13]. Also, several compounds isolated from African plants displayed good inhibitory effects against MDRGN. Amongst these are pomolic acid [14], neobavaisoflavone [15], plumbagin, 4-hydroxylonchocarpin [4] and 5′-methoxyhydnocarpin [16]. The discovery of efflux pump inhibitors (EPIs) is a good alternative to combat MDRGN [17]. EPI generally interact with specific efflux pump proteins to restore the susceptibility of MDR bacteria to antibiotics [18]. The search of EPI phytochemicals that can restore the activity of antibiotics also increase the possibilities to overcome MDR phenotypes. In the past, numbers of plants extracts and derived molecules have been able to potentiate the activity of various classes of antibiotics against MDR bacteria [16, 19–21]. In our continuous quest of naturally occurring bioactive products to tackle bacterial multi-drug resistance, the present study was designed to evaluate the antibacterial activity of methanol extracts and compounds from *Nauclea pobeguinii* (Pobég. ex Pellegr.) Merr. ex E.M.A. (Rubiaceae) against a panel of 29 bacteria including MDR phenotypes. The study was extended to the evaluation of the ability of the studied samples to restore the activity of commonly used antibiotics towards MDR strains. *Nauclea pobeguinii* is used in traditional medicine as abortive and for the treatment of stomach ache, infectious diseases [22], jaundice [23], fever, diarrhea, worm, and malaria [24]. Recently, the plant was shown to have cytotoxic effects on various hematological and carcinoma cell lines [25]. Previous phytochemical investigation of the plant led to the isolation of compounds identified as 3-acetoxy-11-oxo-urs-12-ene (**1**), *p*-coumaric acid (**2**), citric acid trimethyl ester (**3**), resveratrol (**4**), resveratrol *β-*_D_*-*glucopyranoside (**5**) and strictosamide (**6**) [25]. The antimalarial efficacy of stem bark extract of *Nauclea pobeguinii* in human adult volunteers with diagnosed uncomplicated falciparum malaria was also reported [26].

## Methods

### Plant material and extraction

The leaves and bark of *Nauclea pobeguinii* was collected in March and April 2013 at Mbouda (West Region of Cameroon). The plant was identified at the National Herbarium in Yaoundé, Cameroon and compared with voucher formerly kept under the registration number 32597/HNC. Each plant part was air dried and then powdered. The obtained powder (200 g) was extracted with methanol (MeOH; 1 L) for 48 h at room temperature with momentary shaking. Methanol was then removed under reduced pressure to give residues which constituted the crude bark (NPB) and leaves (NPL) extracts. All extracts were then kept at 4 °C until further use.

### Chemicals for antimicrobial assay

**Fig. 1:**
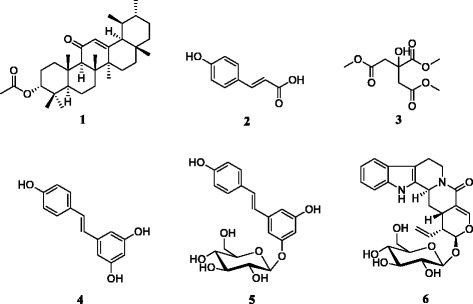
Chemical structures of the compounds isolated from *Nauclea pobeguinii.* **1:** 3-acetoxy-11-oxo-urs-12-ene; **2:** *p*-coumaric acid; **3:** citric acid trimethyl ester; **4:** resveratrol; **5:** resveratrol *β-*_D_ *-*glucopyranoside; **6:** strictosamide

Compounds previously isolated from the bark of *Nauclea pobeguinii* included 3-acetoxy-11-oxo-urs-12-ene (**1**), *p*-coumaric acid (**2**), citric acid trimethyl ester (**3**), resveratrol (**4**), resveratrol *β-*_D_*-*glucopyranoside (**5**) and strictosamide (**6**) (Fig. 1). Their isolation and identification were previously reported [25]. Tetracycline (TET), cefepime (CEP), ciprofloxacin (CIP), chloramphenicol (CHL), ampicillin (AMP), streptomycin (STR), kanamycin (KAN) (Sigma-Aldrich, St Quentin Fallavier, France) were used as reference antibiotics (RA). *p*-Iodonitrotetrazolium chloride (INT; Sigma-Aldrich) and Phenylalanine-Arginine-ß-Naphthylamide (PAßN; Sigma-Aldrich) were used as microbial growth indicator and efflux pumps inhibitor (EPI) respectively [27, 28].

### Microbial strains and culture media

The studied microorganisms included sensitive and resistant strains of *Escherichia coli* (ATTC8739, ATCC10536, AG100, AG100A, AG102, AG100ATet, MC4100, W3110), *Enterobacter aerogenes* (ATCC13048, EA3, EA289, EA294, EA27, EA298, CM64), *Klebsiella pneumoniae* (ATCC11296, KP55, KP63, K2, K24), *Pseudomonas aeruginosa* (PA01, PA124), *Providencia stuartii* (ATCC29914, NEA16, PS299645, PS2636) and *Enterobacter cloacae* (BM47, BM67, ECCI69) obtained clinically or from the American Type Culture Collection (ATCC). Their resistance profiles have been previously reported (see Additional file 1: Table S1). Nutrient agar were used for the activation of the tested Gram-negative bacteria [29].

### INT colorimetric assay for MIC and MBC determinations

The MIC and MBC determinations on the tested bacteria were conducted using rapid *p*-iodonitrotetrazolium chloride (INT) colorimetric assay according to described methods [27] with some modifications [30–32].

To evaluate the antibiotic-potentiating effects, a preliminary study was carried out with 7 antibiotics (AMP, CEF, CHL, CIP, KAN, STR and TET) and samples from *Nauclea pobeguinii* (NPB, NPL, compounds **1–4**) against one of the most probematic bacterial strains, *P. aeruginosa* PA124 (see Additional file 1: Table S2 and S3). Results allowed selecting NPB, NPL and **4** and their antibiotic-potentiating effects were further evaluated. Hence, extracts (NPB and NPL) and compound **4** were tested in association with antibiotics at their sub-inhibitory concentrations (MIC/2 and MIC/4) as obtained in each bacterium [9, 11, 13] respectively against 7 and 6 bacterial strains. Fractional inhibitory concentration (FIC) was calculated as the ratio of MIC_Antibiotic in combination_/MIC_Antibiotic alone_ and the results were discussed as follows: synergy (*≤*0.5), indifferent (>0.5 to 4), or antagonism (*>*4) [33, 34]. All assays were performed in triplicate.

## Results

Compounds tested in this study included 3-acetoxy-11-oxo-urs-12-ene (**1**; purity: 90 %), *p*-coumaric acid (**2**; purity: 97 %), citric acid trimethyl ester (**3**; purity: 97 %), resveratrol (**4**; purity: 98 %), resveratrol *β-*_D_*-*glucopyranoside (**5**; purity: 95 %), and strictosamide (**6**; purity: 96 %) previously isolated in the bark of *Nauclea pobeguinii* [25]. The antibacterial activity of these compounds as well as the crude extracts was evaluated in a panel of Gram-negative bacteria including MDR phenotypes. The results are summarized in Tables 1 and 2.

**Table 1:** MIC and MBC (μg/mL) of *Nauclea pobeguinii* extracts and chloramphenicol on the panel of tested bacteria

Bacterial strains	Tested samples, MIC and MBC (μg/mL)		
	NPB	NPL	CHL
*Escherichia coli*			
ATCC8739	512 (>1024)	128 (>1024)	**8** (256)
ATCC10536	**32** (1024)	256 (>1024)	16 (32)
W 3110	1024 (>1024)	>1024	64 (128)
MC4100	256 (>1024)	256 (>1024)	128 (128)
AG100 A	1024 (>1024)	1024 (>1024)	64 (64)
AG100Atet	256 (256)	512 (1024)	64 (128)
AG102	1024 (>1024)	512 (>1024)	64 (64)
AG100	**64** (256)	512 (>1024)	16 (64)
*Enterobacter aerogenes*			
ATCC13048	1024 (>1024)	1024 (>1024)	**8** (32)
EA294	1024 (>1024)	>1024	16 (128)
CM64	1024 (>1024)	>1024	128 (>256)
EA298	**64** (1024)	1024 (1024)	256 (>256)
EA27	512 (>1024)	256 (>1024)	>256
EA289	>1024	1024 (>1024)	256 (>256)
EA3	512 (>1024)	1024 (>1024)	>256
*Klebsiella pneumoniae*			
ATCC11296	256 (>1024)	256 (>1024)	**8** (256)
KP55	128 (>1024)	128 (>1024)	32 (128)
KP63	1024 (>1024)	256 (>1024)	128 (>256)
K2	512 (>1024)	>1024	64 (256)
K24	1024 (>1024)	512 (>1024)	32 (256)
*Pseudomonas aeruginosa*			
PA01	>1024	1024 (>1024)	128 (>256)
PA124	1024 (>1024)	>1024	256 (>256)
*Providencia stuartii*			
ATCC29916	1024 (>1024)	>1024	16 (32)
PS2636	512 (1024)	>1024	32 (32)
PS299645	1024 (>1024)	512 (1024)	32 (256)
NEA16	512 (>1024)	512 (>1024)	256 (>256)
*Enterobacter cloacae*			
BM47	1024 (>1024)	>1024	256 (>256)
ECCI69	256 (512)	512 (1024)	>256
BM67	>1024	>1024	256 (>256)

The Tested extract were obtained from the bark (NPB) and leaves (NPL); Values in bold; significant activity

*CHL* chloramphenicol, *MIC* Minimal Inhibitory Concentration, *MBC* Minimal Bactericidal Concentration

**Table 2:** MICs and MBCs (μg/mL) of compounds from *Nauclea pobeguinii* against selected bacterial strains

Bacterial strains	Compounds, MIC and MBC (in bracket)					
	1	2	3	4	5	6
*Escherichia coli*						
ATCC8739	256 (>256)	>256	>256	64 (64)	>256	>256
AG100Atet	>256	>256	>256	128 (256)	>256	>256
AG102	>256	>256	1024 (>256)	32 (32)	>256	>256
*Enterobacter aerogenes*						
CM64	>256	>256	>256	64 (>256)	>256	>256
*Klebsiella pneumoniae*						
ATCC11296	>256	>256	>256	64 (>256)	>256	>256
KP55	>256	>256	32 (128)	16 (32)	>256	256 (>256)
*Pseudomonas aeruginosa*						
PA01	>256	>256	>256	64 (128)	>256	256 (>256)
PA124	>256	>256	>256	256 (>256)	>256	>256
*Providencia stuartii*						
ATCC29916	>256	>256	>256	128 (>256)	>256	>256
PS2636	>256	>256	>256	256 (>256)	>256	>256

**1:** 3-acetoxy-11-oxo-urs-12-ene; **2:** *p*-coumaric acid; **3:** citric acid trimethyl ester ; **4:** resveratrol; **5:** resveratrol *β-*_D_ *-*glucopyranoside; **6:** strictosamide

MIC results as compiled in Table 1 indicate that values ranging from 32-1024 μg/mL were obtained with NPB and NPL respectively on 26/29 (89.7 %) and 20/29 (69.0 %) of the tested bacterial strains. The lowest MIC value of 32 μg/mL was recorded with NPB against *Escherichia coli* ATCC10536. Table 2 reports the MIC values of compounds from NPB. It’s appears that **4** was active on all the ten selected bacteria including ATCC strains and clinical MDR phenotypes, whilst **1**, **3** and **6** displayed poor and selective inhibitory effects. Compounds **2** and **5** were not active on all tested microorganisms. The lowest MIC value for compound (16 μg/mL) was obtained with **4** against *Klebsiella pneumoniae* KP55 strain. The best extract (NPB) as well as the most active compound (**4**) had low bactericidal activities, displaying MBC values only against 6/29 (20.7 %) and 5/10 (50 %) of the tested pathogens respectively.

Seven antibiotics used in bacterial chemotherapy were combined with NPB, NPL, compounds **1–4** and tested in a preliminary study against the problematic nosocomial pathogen, *P. aeruginosa* PA124. The results (Additional file 1: Table S2 and S3) indicated that there was no improvement of the activity of the two beta-lactamines tested (CEF and AMP). Consequently, CEF and AMP as well as compounds **1**–**3** having low antibiotic-potentiating activity against PA124 were not further investigated. Five antibiotics (CIP, TET, KAN, STR and CHL) were combined with NPB, NPL and compound **4** at their MIC/2 and MIC/5, as obtained on each of the tested bacterial strains (Tables 3 and 4). At MIC/2 of the samples, NPB and **4** (6/6) had synergistic effects with CHL and KAN on 100 % tested MDR bacteria. At MIC/4, 100 % synergistic effects were also obtained when NPB was combined with STR (Table 3) and when **4** was associated with STR and CIP (Table 4). NPL had lower synergistic effects with antibiotic at MIC/2 and MIC/4.

**Table 3:** MIC of antibiotics in association of bark and leaves extracts of *Nauclea pobeguinii* at MIC/2 and MIC/4 against selected MDR bacteria

Antibiotics^a^	Extract and concentration		Bacterial strains^b^, MIC (μg/mL ) of antibiotics in the absence and presence of the extract and FIC (in brackets)							PBSS (%)
			PA124	CM64	NAE16	BM47	KP55	KP63	AG100Atet	
CHL		0	**256**	**128**	**256**	**256**	**32**	**128**	**64**	
	B	MIC/2	128(0.5)S	64(0.5)S	32(0.125)S	16(0.06)S	16(0.5)S	8(0.06)S	32(0.5)S	**7/7 (100 %)**
		MIC/4	128(0.5)S	64(0.5)S	32(0.13)S	16(0.06)S	32(1)I	8(0.06)S	32(0.5)S	**6/7 (85.7 %)**
	L	MIC/2	256(1)I	128(1)I	16(0.06)S	32(0.13)S	32(1)I	8(0.06)S	16(0.25)S	4/7 (57.1)
		MIC/4	256(1)I	128(1)I	16(0.06)S	64(0.25)S	32(1)I	8(0.06)S	16(0.25)S	4/7 (57.1)
KAN		0	**128**	**64**	**64**	**32**	**64**	**64**	**32**	
	B	MIC/2	64(0.5)S	4(0.06)S	8(0.13)S	4(0.13)S	32(0.5)S	8(0.13)S	16(0.5)S	**7/7 (100 %)**
		MIC/4	64(0.5)S	4(0.06)S	8(0.13)S	4(0.13)S	32(0.5)S	8(0.13)S	16(0.5)S	**7/7 (100 %)**
	L	MIC/2	64(0.5)S	4(0.06)S	8(0.13)S	4(0.13)S	64(1)I	16(0.25)S	16(0.5)S	**6/7 (85.7 %)**
		MIC/4	64(0.5)S	4(0.06)S	8(0.13)S	4(0.13)S	64(1)I	16(0.25)S	16(0.5)S	**6/7 (85.7 %)**
STR		0	**64**	**64**	**32**	**16**	**64**	**16**	**128**	
	B	MIC/2	64(1)I	4(0.06)S	16(0.5)S	4(0.25)S	32(0.5)S	2(0.13)S	−(≥2)na	**5/6 (83.3 %)**
		MIC/4	256(4)A	4(0.06)S	16(0.5)S	4(0.25)S	32(0.5)S	8(0.5)S	−(≥2)na	**5/6 (83.3 %)**
	L	MIC/2	256(4)A	2(0.03)S	8(0.25)S	2(0.13)S	16(0.25)S	4(0.25)S	128(1)I	**5/7 (71.4 %)**
		MIC/4	256(4)A	4(0.06)S	16(0.5)S	4(0.25)S	64(1)I	4(0.25)S	−(≥2)na	4/6 (66.7 %)
CIP		0	**64**	**16**	**8**	**16**	**16**	**8**	**64**	
	B	MIC/2	32(0.5)S	64(4)A	8(1)I	8(0.5)S	16(1)I	4(0.5)S	64(1)I	3/7 (42.9 %)
		MIC/4	32(0.5)S	64(4)A	8(1)I	16(1)I	32(2)A	8(1)I	64(1)I	1/7 (14.3 %)
	L	MIC/2	64(1)I	64(4)A	16(2)A	16(1)I	16(1)I	4(0.5)S	64(1)I	1/7 (14.3 %)
		MIC/4	64(1)I	64(4)A	16(2)A	16(1)I	16(1)I	4(0.5)S	64(1)I	1/7 (14.3 %)
TET		0	**64**	**64**	**128**	**32**	**32**	**64**	**256**	
	B	MIC/2	32(0.5)S	4(0.06)S	32(0.25)S	16(0.5)S	64(2)I	32(0.5)S	4(0.02)S	**6/7 (85.7 %)**
		MIC/4	32(0.5)S	2(0.03)S	32(0.25)S	16(0.5)S	64(2)I	64(1)I	4(0.02)S	**5/7 (71.4 %)**
	L	MIC/2	64(1)I	2(0.03)S	128(1)I	32(1)I	−(≥8)A	64(1)I	4(0.02)S	2/7 (28.6 %)
		MIC/4	64(1)I	2(0.03)S	128(1)I	32(1)I	−(≥8)A	64(1)I	4(0.02)S	2/7 (28.6 %)

(−): >256 μg/mL; 0: no extract (only antibiotic tested); na: non-applicable; Values in bold: MIC of antibiotic alone and significant synergistic effects

*S* Synergy; *I* Indifference, *A* Antagonism, *B* bark extract, *L* Leaves extract, *FIC* fractional inhibitory concentration

^a^Antibiotics [CHL: chloramphenicol, AMP : ampicillin, CEF: cefepime, KAN : kanamycin, STR: streptomycin, CIP : ciprofloxacin, TET : tetracycline]

^b^Bacterial strains: *Escherichia coli* [ AG100Atet], *Pseudomonas aeruginosa* [PA124], *Enterobacter aerogenes* [CM64], *Enterobacter cloacae* [BM47], *Klebsiella pneumoniae* [KP55, KP63], *Providencia stuartii* [NAE16]

^c^PBSS: percentage of bacteria strain on which synergism has been observed; (): fold increase in MIC values of the antibiotics after association with plants extract

**Table 4:** MIC of antibiotics after the association of resveratrol (**4**) at MIC/2 and MIC/4 against selected MDR bacteria

Antibiotics^a^	Concentration of 4	Bacteria strains^b^, MIC of antibiotics alone and in presence of 4 (Resveratrol), FIC (in bracket )						PBSS (%)^c^
		PA124	CM64	KP55	AG102	AG100Atet	PS2636	
**CHL**	**0**	**256**	**128**	**32**	**64**	**64**	**256**	
	MIC/2	32 (0.25)S	2(0.02)S	16(0.5)S	64(1)I	**<2(<**0.03)S	64(0.25)S	**5/6 (83.3 %)**
	MIC/4	32(0.25)S	4(0.03)S	16(0.5)S	64(1)I	**<2 (<**0.03)S	64(0.25)S	**5/6(83.3 %)**
**KAN**	**0**	**128**	**64**	**64**	**4**	**32**	**4**	
	MIC/2	64(0.5)S	<2 (<0.03)S	32(0.5)S	<2 (<0.5)S	<2(0.06)S	4(1)I	**5/6(83.3 %)**
	MIC/4	64(0.5)S	<2 (<0.03)S	32(0.5)S	<2 (<0.5)S	<2(0.06)S	4(1)I	**5/6(83.3 %)**
**STR**	**0**	**64**	**128**	**64**	**≤2**	**−**	**≤2**	
	MIC/2	32(0.5)S	64(0.5)S	16(0.25)S	<2 na	64(≤0.5)S	<2 na	**4/4 (100 %)**
	MIC/4	32(0.5)S	64(0.5)S	32(0.5)S	4(>2) na	128(≤0.5)S	<2 na	**4/4 (100 %)**
**CIP**	**0**	**64**	**8**	**4**	**1**	**64**	**4**	
	MIC/2	4(0.06)S	**<2**(0.25)S	**<2**(<0.5)S	<0.5(<0.5)S	**<2(<**0.03)S	8(2)I	**5/6(83.3 %)**
	MIC/4	16(0.25)S	**<2**(0.25)S	**<2**(<0.5)S	<0.5(<0.5)S	**<2(<**0.03)S	<0.5(<0.13)S	**6/6 (100 %)**
**TET**	**0**	**64**	**64**	**32**	**4**	**256**	**64**	
	MIC/2	4(0.06)S	64(1)I	32(1)I	**<2**(<0.5)S	256(1)I	32(0.5)S	3/6 (50 %)
	MIC/4	4(0.06)S	128(2)I	16(0.5)S	**<2**(<0.5)S	256(1)I	16(0.25)S	4/6 (66.7 %)

(−): >256 μg/mL; 0: without compound 4 (only antibiotic tested). CIP have been tested at 64 μg/ml on AG102 and PS2636; na: non-applicable; Values in bold: MIC of antibiotic alone or significant synergistic effects

*S* Synergy, *I* Indifference, *A* Antagonism, *P* product, *FIC* fractional inhibitory concentration

^a^Antibiotics [CHL: chloramphenicol, KAN : kanamycin, STR: streptomycin, CIP : ciprofloxacin, TET : tetracycline]

^b^Bacterial strains: *Escherichia coli* [ AG102, AG100Atet], *Pseudomonas aeruginosa* [PA124], *Enterobacter aerogenes* [CM64], *Klebsiella pneumoniae* [KP55], *Providencia stuartii* [PS2636]

^c^PBSS: percentage of bacteria strain on which synergism has been observed

## Discussion

MDR resistant bacteria of the family enterobacteriaceae or as well as the nosocomial pathogen *Pseudomonas aeruginosa* are largely involved clinically in treatment failures [35]. Clinical bacteria used in the present study actively express efflux pumps [5, 9, 35, 36] and therefore represent good models in the search of chemicals to combat drug resistance. Phytochemicals are routinely classified as significantly active antibacterial agents on the basis of their MIC values below 100 μg/mL for crude extracts or 10 μg/mL for compounds; the activity is considered moderate when 100 < MIC < 625 μg/mL (crude extracts) or 10 < MIC < 100 μg/mL for molecules [37–39]. Taking in account these cutoff points, it can be deduced that NPB had a good antibacterial potential, as MIC values below 100 μg/mL were obtained with this extract against *E. coli* ATCC10536, AG100 and *Enterobacter aerogenes* CM64 strains. In addition, the MIC value of 64 μg/mL obtained with NPB against *E. aerogenes* EA298 strain was lower than that of the reference drug CHL (256 μg/mL). Nonetheless, compounds **1–6** (from NPB) rather had moderate, low or no inhibitory effects, suggesting that they may act synergistically in NPB. However, the lowest MIC value of 16 μg/mL obtained with **4** was better than that of CHL (32 μg/mL) against *K. pneumoniae* KP55 strain, also highlighting the possible usefulness of this compound in the fight against MDR bacteria. It is worth noting that compound **5** (a glucoside of **4)** was not active contrary to its aglycon **4**, indicating that the presence of glucose in compound **5** significantly reduces its antibacterial activities.

Reversal of multi-drug resistance appears today as another attempt to mitigate the spread of resistance in bacteria. In recent years, many botanicals showed antibiotic-modulation effect in efflux pumps over-expressing MDR bacteria [9, 10, 16, 19, 20, 40–42]. In the present study, we observed that a beneficial effects of the combination of NPB with CHL, KAN as well as that of compound **4** with STR and CIP in all tested bacteria were achieved. Synergistic or modulating effects of NPB and **4** with other antibiotics were noted on more than 70 % of the tested MDR bacteria in several case (Tables 3 and 4), suggesting that they can act as efflux pump inhibitors [40]. This is strenghten by the fact that no synergistic effect was obtained with beta-lactamines (CEF and AMP) in the preliminary test (Additional file 1: Tables S2 and S3), as their target are located in the bacterial coat and hence, are not generally affected by AcrAB-TolC and MexAB-OprM efflux pumps in Enterobacteriaceae and *P. aeruginosa* respectively [4].

This is the first time to report the potential of NPB to prevent the proliferation of MDR Gram-negative bacterial as well as to reverse antibiotic resistance in MDR bacteria. However, the methanol extract from roots of *Nauclea pobeguinii* showed synergistic effects with ampicillin and amoxicillin against *Staphylococcus aureus* and drug-sensitive *Klebsiella pneumoniae* [43]. The present study therefore provides additional information on the ability of other parts of *Nauclea pobeguinii* to potentiate the activity of antibiotics. Though, the antibacterial potential of compound **4** is well known [44], the present study also identify this stilbene as the potent antibacterial constituent of *Nauclea pobeguiinii.* This study also provides more information on its inhibitory potential against MDR bacteria expressing active efflux pumps as well as it ability to potentiate the activity of antibiotics.

## Conclusion

The results reported herein are very interesting, in regards to the medical importance of the studied microorganisms. These data provide evidence that crude extracts and compounds from *Nauclea pobeguinii* and mostly the bark extract (NPB) and compound **4** are potential sources of compounds to fight MDR bacterial species. The bark extract and **4** could also be used in combination with antibiotics to overcome bacterial resistance.

## Abbreviations

**1**, 3-acetoxy-11-oxo-urs-12-ene; **2**, *p*-coumaric acid; **3**, citric acid trimethyl ester; **3**, resveratrol; **4**, resveratrol *β-*_D_*-*glucopyranoside; **6**, strictosamide; AMP, ampicillin; ATCC, American Type Culture Collection; CEF, cefepime; CHL, chloramphenicol; CIP, ciprofloxacin; EPI, efflux pump inhibitors; FIC, fractional inhibitory concentration; HNC, National Herbarium of Cameroon; INT, *p*-iodonitrotetrazolium chloride; KAN, kanamycin; MBC, minimal bactericidal concentrations; MDR, multidrug resistant; MDRGN, multidrug resistant Gram-negative; MeOH, methanol; MIC, minimal inhibitory concentrations; NPB, *Nauclea pobeguiinii* bark; NPL, *Nauclea pobeguiinii* leaves; RA, reference antibiotics; RND, resistance–nodulation–cell division; STR, streptomycin; TET, Tetracycline.

## Additional file

Additional file 1: Table S1. Bacterial strains used and their features. **Table S2.** Preliminary assay of extracts from bark and leaves of *Nauclea pobeguinii* in combination with commonly used antibiotics against PA124. **Table S3.** Preliminary assay with compounds in combination with commonly used antibiotics against PA124. (DOC 173 kb)
