# Supplementary material for: Antibacterial and antibiotic-resistance modifying activity of the extracts and compounds from Nauclea pobeguinii against Gram-negative multi-drug resistant phenotypes
Source: BMC Complement Altern Med. 2016 Jul 7;16:193. doi: 10.1186/s12906-016-1173-2 (PMC4937598; doi:10.1186/s12906-016-1173-2)
Supplement: Additional file 1: Table S1. — Bacterial strains used and their features. Table S2. Preliminary assay of extracts from bark and leaves of Nauclea pobeguinii in combination with commonly used antibiotics against PA124. Table S3. Preliminary assay with compounds in combination with commonly used antibiotics against PA124. (DOC 173 kb) [file 12906_2016_1173_MOESM1_ESM.doc]

**Antibacterial and Antibiotic Resistance Modifying Activity of the Extracts and compounds from *Nauclea pobeguinii* against Gram-negative Multi-Drug Resistant Phenotypes**

Jackson A. Seukep^1^, Louis P. Sandjo^2^, Bonaventure T. Ngadjui^3^ and Victor Kuete^1*^

*^1^Department of Biochemistry, Faculty of Science, University of Dschang, Cameroon;*

*^2^Department of Pharmaceutical Sciences, CCS, Federal University of Santa Catarina, Florianópolis, 88040-900, SC, Brazil;*

*^3^Department of Organic Chemistry, Faculty of Science, University of Yaoundé 1, Cameroon.*

**Corresponding author:**

**Tel:(+237)677355927; E-mail: kuetevictor@yahoo.fr (Prof. Dr. Victor Kuete)*

**Authors e-mails:**

Jackson A. Seukep: *seukepp@yahoo.fr*

Louis P. Sandjo: *plsandjo@yahoo.fr*

Bonaventure T. Ngadjui: *btngadjui@yahoo.fr*

Victor Kuete: *kuetevictor@yahoo.fr*

**Table S1.** Bacterial strains used and their features

| Strains | Features and References |  |
| --- | --- | --- |
| *Escherichia coli* |  |  |
| ATCC10536 and ATCC8739 | Reference strain |  |
| AG100 | Wild-type *E. coli* K-12 | [1] |
| AG100A | AG100 *ΔacrAB*::KAN^R^ | [1-3] |
| AG100A_TET_ | Δ*acrAB* mutant AG100, with over-expressing  *acrF* gene ; TET^R^ | [1] |
| AG102 | Δ*acrAB* mutant AG100, owing *acrF* gene markedly over-expressed; TET^R^ | [4, 5] |
| MC4100 | Wild type *E. coli* | [6] |
| W3110 | Wild type *E. coli* | [6, 7] |
| *Enterobacter aerogenes* |  |  |
| ATCC13048 | Reference strains |  |
| CM64 | CHL^R^ resistant variant obtained from ATCC13048 over-expressing the AcrAB pump | [8] |
| EA3 | Clinical MDR isolate; CHL^R^, NOR^R^, OFX^R^, SPX^R^, MOX^R^, CFT^R^, ATM^R^, FEP^R^ | [9, 10] |
| EA27 | Clinical MDR isolate exhibiting energy-dependent norfloxacin and chloramphenicol efflux with KAN^R^ AMP^R^ NAL^R^ STR^R^ TET^R^ | [9, 10] |
| EA289 | KAN sensitive derivative of EA27 | [11] |
| EA294 | EA289 a*crA::*KAN^R^ | [11] |
| EA298 | EA 289 *tolC::*KAN^R^ | [11] |
| *Enterobacter cloacae* |  |  |
| ECCI69 | Clinical MDR isolates, CHL^R^ | [12] |
| BM67 | Clinical MDR isolates, CHL^R^ | [12] |
| BM47 | Clinical MDR isolates, CHL^R^ | [12] |
| *Klebsiella pneumoniae* |  |  |
| ATCC12296 | Reference strains |  |
| KP55 | Clinical MDR isolate, TET^R^ , AMP^R^, ATM^R^, CEF^R^ | [13] |
| KP63 | Clinical MDR isolate, TET^R^, CHL^R^, AMP^R^, ATM^R^ | [13] |
| K24 | AcrAB-TolC, Laboratory collection of UNR-MD1, University of Marseille, France | [12] |
| K2 | AcrAB-TolC, Laboratory collection of UNR-MD1, University of Marseille, France | [12] |
| *Providencia stuartii* |  | [14] |
| ATCC29916 | Reference strain |
| NEA16 | Clinical MDR isolate, AcrAB-TolC |
| PS2636 | Clinical MDR isolate, AcrAB-TolC |
| PS299645 | Clinical MDR isolate, AcrAB-TolC |
| *Pseudemonas aeruginosa* |  |  |
| PA 01 | Reference strains |  |
| PA 124 | MDR clinical isolate | [15] |

^a^AMP, ATM^R^, CEF^R^, CFT^R^, CHL^R^, FEP^R^, KAN^R^, MOX^R^, OFX^R^, STR^R^, TET^R^. Resistance to ampicillin, aztreonam, cephalothin, cefadroxil, chloramphenicol, cefepime, kanamycin, moxalactam, ofloxacin, streptomycin, and tetracycline; MDR : Multidrug resistant; AcrAB-TolC efflux pump AcrAB associate to TolC porin,

**Table S2.**Preliminary assay of extracts from bark and leaves of *Nauclea pobeguinii*  in combination with commonly used antibiotics against PA124

| **Antibiotics** | **MIC of antibiotic alone** | **MIC (** **µg/mL) of antibiotic in the presence of extract, FIC ( in bracket)** | | | |
| --- | --- | --- | --- | --- | --- |
|  |  | **NPB** | | **NPL** | |
|  |  | **MIC/2** | **MIC/4** | **MIC/2** | **MIC/4** |
| **CHL** | **256** | 128(0.5)S | 128(0.5)S | 256(1)I | 256(1)I |
| **AMP** | **-** | **-** | **-** | **-** | **-** |
| **CEF** | **-** | **-** | **-** | **-** | **-** |
| **KAN** | **128** | 64(0.5)S | 64(0.5)S | 64(0.5)S | 64(0.5)S |
| **STR** | **64** | 64(1)I | 64(1)I | 256(4)A | 256(4)A |
| **CIP** | **64** | 32(0.5)S | 32(0.5)S | 64(1)I | 64(1)I |
| **TET** | **64** | 32(0.5)S | 32(0.5)S | 64(1)I | 64(1)I |

NPB: Bark extract; NPL: leaves extract; S: Synergy, I: Indifference; A: Antagonism; FIC: fractional inhibitory concentration; (-): >256 µg/mL. ATB: Antibiotics; CHL: chloramphenicol; AMP: ampicillin; CEF: cefepime; KAN: kanamycine; STR: streptomycin; CIP: ciprofloxacin; TET: tetracycline;

**Table S3**. Preliminary assay with compounds in combination with commonly used antibiotics against PA124

| **Antibiotics** | **Concentration of compound from NPB** |  | **MIC of antibiotic alone and in presence of compounds, FIC (in bracket)** | | |
| --- | --- | --- | --- | --- | --- |
|  |  | **1** | **2** | **3** | **4** |
| **CHL** | 0 | **256** | **256** | **256** | **256** |
|  | MIC/2 | 64(0.25)S | 64(0.25)S | 64(0.25)S | 32 (0.25)S |
|  | MIC/4 | 64(0.25)S | 64(0.25)S | 64(0.25)S | 32(0.25)S |
| **AMP** | 0 | **-** | **-** | **-** | **-** |
|  | MIC/2 | **-** | **-** | **-** | - |
|  | MIC/4 | **-** | **-** | **-** | - |
| **CEF** | 0 | **-** | **-** | **-** | - |
|  | MIC/2 | **-** | **-** | **-** | - |
|  | MIC/4 | **-** | **-** | **-** | - |
| **KAN** | 0 | **128** | **128** | **128** | **128** |
|  | MIC/2 | 128(1)I | 128(1)I | 128(1)I | 64(0.5)S |
|  | MIC/4 | 128(1)I | 128(1)I | 128(1)I | 64(0.5)S |
| **STR** | 0 | **64** | **64** | **64** | **64** |
|  | MIC/2 | 32(0.5)S | 64(1)I | 64(1)I | 32(0.5)S |
|  | MIC/4 | 32(0.5)S | 64(1)I | 64(1)I | 32(0.5)S |
| **CIP** | 0 | **64** | **64** | **64** | **64** |
|  | MIC/2 | 16(0.25)S | 16(0.25)S | 64(1)I | 4(0.06)S |
|  | MIC/4 | 16(0.25)S | 16(0.25)S | 64(1)I | 16(0.25)S |
| **TET** | 0 | **64** | **64** | **64** | **64** |
|  | MIC/2 | 8(0.13)S | 8(0.13)S | 8(0.13)S | 4(0.06)S |
|  | MIC/4 | 8(0.13)S | 8(0.13)S | 8(0.13)S | 4(0.06)S |

S: Synergy, I: Indifference; A: Antagonism; FIC: fractional inhibitory concentration; (-): >256 µg/mL; 0: no extract (only antibiotic tested). CHL: chloramphenicol; AMP: ampicillin; CEF: cefepime; KAN: kanamycine; STR: streptomycin; CIP: ciprofloxacin; TET: tetracycline; **1**: 3-acetoxy-11-oxo-urs-12-ene; **2**: *p*-coumaric acid; **3:** citric acid trimethyl ester; **4**: resveratrol; NPB: *Nauclea pobeguinii* bark.

**References**

1. Viveiros M, Jesus A, Brito M, Leandro C, Martins M, Ordway D, Molnar AM, Molnar J, Amaral L. Inducement and reversal of tetracycline resistance in Escherichia coli K-12 and expression of proton gradient-dependent multidrug efflux pump genes. *Antimicrob Agents Chemother* 2005, 49(8):3578-3582.

2. Okusu H, Ma D, Nikaido H. AcrAB efflux pump plays a major role in the antibiotic resistance phenotype of Escherichia coli multiple-antibiotic-resistance (Mar) mutants. *J Bacteriol* 1996, 178(1):306-308.

3. Kuete V, Ngameni B, Tangmouo JG, Bolla JM, Alibert-Franco S, Ngadjui BT, Pages JM. Efflux pumps are involved in the defense of Gram-negative bacteria against the natural products isobavachalcone and diospyrone. *Antimicrob Agents Chemother* 2010, 54(5):1749-1752.

4. Elkins CA, Mullis LB. Substrate competition studies using whole-cell accumulation assays with the major tripartite multidrug efflux pumps of *Escherichia coli*. *Antimicrob Agents Chemother* 2007, 51(3):923-929.

5. Kuete V, Alibert-Franco S, Eyong KO, Ngameni B, Folefoc GN, Nguemeving JR, Tangmouo JG, Fotso GW, Komguem J, Ouahouo BM *et al*. Antibacterial activity of some natural products against bacteria expressing a multidrug-resistant phenotype. *Int J Antimicrob Agents* 2011, 37(2):156-161.

6. Baglioni P, Bini L, Liberatori S, Pallini V, Marri L. Proteome analysis of *Escherichia coli* W3110 expressing an heterologous sigma factor. *Proteomics* 2003, 3(6):1060-1065.

7. Sar C, Mwenya B, Santoso B, Takaura K, Morikawa R, Isogai N, Asakura Y, Toride Y, Takahashi J. Effect of *Escherichia coli* wild type or its derivative with high nitrite reductase activity on in vitro ruminal methanogenesis and nitrate/nitrite reduction. *J Anim Sci* 2005, 83(3):644-652.

8. Ghisalberti D, Masi M, Pages JM, Chevalier J. Chloramphenicol and expression of multidrug efflux pump in *Enterobacter aerogenes*. *Biochem Biophys Res Commun* 2005, 328(4):1113-1118.

9. Mallea M, Chevalier J, Bornet C, Eyraud A, Davin-Regli A, Bollet C, Pages JM. Porin alteration and active efflux: two in vivo drug resistance strategies used by *Enterobacter aerogenes*. *Microbiology* 1998, 144 ( Pt 11):3003-3009.

10. Mallea M, Mahamoud A, Chevalier J, Alibert-Franco S, Brouant P, Barbe J, Pages JM. Alkylaminoquinolines inhibit the bacterial antibiotic efflux pump in multidrug-resistant clinical isolates. *Biochem J* 2003, 376(Pt 3):801-805.

11. Pradel E, Pages JM. The AcrAB-TolC efflux pump contributes to multidrug resistance in the nosocomial pathogen *Enterobacter aerogenes*. *Antimicrob Agents Chemother* 2002, 46(8):2640-2643.

12. Fankam AG, Kuete V, Voukeng IK, Kuiate JR, Pages JM. Antibacterial activities of selected Cameroonian spices and their synergistic effects with antibiotics against multidrug-resistant phenotypes. *BMC Complement Altern Med* 2011, 11:104.

13. Chevalier J, Pages JM, Eyraud A, Mallea M. Membrane permeability modifications are involved in antibiotic resistance in *Klebsiella pneumoniae*. *Biochem Biophys Res Commun* 2000, 274(2):496-499.

14. Tran QT, Mahendran KR, Hajjar E, Ceccarelli M, Davin-Regli A, Winterhalter M, Weingart H, Pages JM. Implication of porins in beta-lactam resistance of *Providencia stuartii.* *J Biol Chem* 2010, 285(42):32273-32281.

15. Lorenzi V, Muselli A, Bernardini AF, Berti L, Pages JM, Amaral L, Bolla JM. Geraniol restores antibiotic activities against multidrug-resistant isolates from gram-negative species. *Antimicrob Agents Chemother* 2009, 53(5):2209-2211.
